# Epigenetic reprogramming during the maternal‐to‐zygotic transition

**DOI:** 10.1002/mco2.331

**Published:** 2023-08-02

**Authors:** Yurong Chen, Luyao Wang, Fucheng Guo, Xiangpeng Dai, Xiaoling Zhang

**Affiliations:** ^1^ Key Laboratory of Organ Regeneration and Transplantation of Ministry of Education First Hospital of Jilin University Changchun China; ^2^ National‐Local Joint Engineering Laboratory of Animal Models for Human Disease First Hospital of Jilin University Changchun China

**Keywords:** chromatin, embryo development, epigenetics, MZT, reprogramming, ZGA

## Abstract

After fertilization, sperm and oocyte fused and gave rise to a zygote which is the beginning of a new life. Then the embryonic development is monitored and regulated precisely from the transition of oocyte to the embryo at the early stage of embryogenesis, and this process is termed maternal‐to‐zygotic transition (MZT). MZT involves two major events that are maternal components degradation and zygotic genome activation. The epigenetic reprogramming plays crucial roles in regulating the process of MZT and supervising the normal development of early development of embryos. In recent years, benefited from the rapid development of low‐input epigenome profiling technologies, new epigenetic modifications are found to be reprogrammed dramatically and may play different roles during MZT whose dysregulation will cause an abnormal development of embryos even abortion at various stages. In this review, we summarized and discussed the important novel findings on epigenetic reprogramming and the underlying molecular mechanisms regulating MZT in mammalian embryos. Our work provided comprehensive and detailed references for the in deep understanding of epigenetic regulatory network in this key biological process and also shed light on the critical roles for epigenetic reprogramming on embryonic failure during artificial reproductive technology and nature fertilization.

## Graphical abstract

Graphical Abstract: The DNA methylation, histone modification, chromatin structure, and RNA modification are important epigenetic modifications that are reprogrammed during the maternal‐to‐zygote transition (MZT). Their reprogramming processes are regulated by multiple underlying molecular mechanisms that were discussed in the paper. The candidate regulators, such as the RNA methylation, BTG4, CCR4‐NOT, tut4/7, and NR5A2, are involved in the maternal mRNAs degradation and zygotic genome activation initiation during MZT. Therefore, it provided detailed and comprehensive resources for further investigation on epigenetic regulatory network during this key biological process.

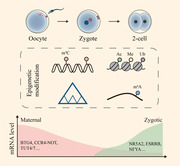

## 1 INTRODUCTION

Zygote is the first form of a new life in sexual organisms. At the developmental stage, both parental genomes of the zygote must be reprogrammed to accomplish the transition from terminally differentiated state to totipotency state.^1^ At this stage, the control of embryonic development transfers from the maternal components to the zygotic genome, a process termed maternal‐to‐zygotic transition (MZT).^2^ MZT includes two major events, the widespread transcriptional activation of the zygotic genome as well as the degradation of accumulated maternal mRNAs and proteins during oocyte maturation.^2^, ^3^ Maternal mRNAs are decayed through both maternal‐factor‐mediated (M‐decay) and zygotic‐factor‐mediated (Z‐decay) pathways.^4^ In mouse embryos, maternally inherited mRNAs are mostly eliminated by the end of the 2‐cell stage and completely eliminated at the 4‐cell stage.^5^, ^6^ Moreover, the maternal proteins are quickly degraded after fertilization.^7^ In human embryos, the majority of maternal mRNAs are eliminated at the 8‐cell stage.^8^

**FIGURE 1:**
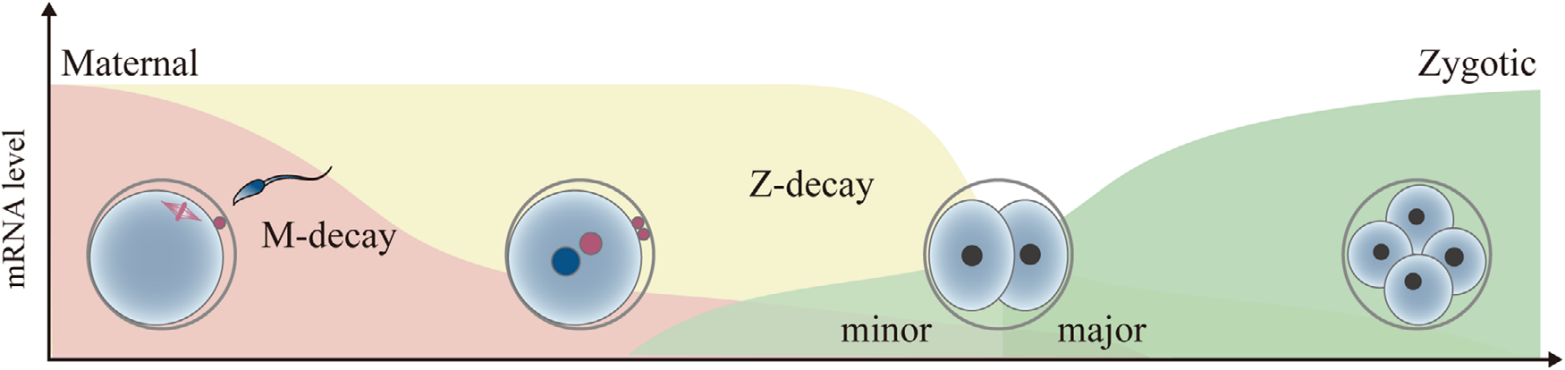
**Dynamics of transcriptome during early mouse embryo development**. Maternal mRNAs undergo degradation after fertilization, depending on maternal‐factor‐mediated (M‐decay) and zygotic‐factor‐mediated (Z‐decay) pathways. The zygotic genome activation (ZGA) occurs in two successive waves: the minor ZGA and major ZGA.

In addition, zygotic genome activation (ZGA) occurs gradually after fertilization, which is a process that both maternal and paternal genomes begin to be activated in a temporally controllable manner.^9^ The timing of ZGA varies in different species. In mice embryos, ZGA occurs in two successive waves: the minor ZGA that occurs between the S phase of the 1‐cell stage and the G1 of the 2‐cell stage, and the major ZGA that occurs during the mid‐to‐late 2‐cell stage.^10^, ^11^ Minor ZGA is characterized by low level of genome‐wide and promiscuous ectopic transcription that produces inefficiently spliced and polyadenylated transcripts. However, during subsequent major ZGA, thousands of genes started to transcribe with functional posttranscriptional processing (Figure 1).^12^ In human embryos, gene expression initiates at the 1‐cell stage and major ZGA mainly starts around 8‐cell stage.^13^ Furthermore, a recent study found that the major ZGA seems to be first initiated from the paternal genome in human 8‐cell embryos.^14^

Epigenetic modifications which could regulate the gene expression without alterations in DNA sequence include DNA/RNA modifications, post‐translational modifications of histone, and three‐dimensional (3D) chromatin organization.^15^, ^16^, ^17^ Different epigenetic modifications work closely together and constitute an epigenome regulatory network, which play a pivotal role in the maintenance of cell identity and function.^18^, ^19^, ^20^, ^21^ During MZT, embryos undergo the transition from a differentiated state to a totipotent state, and various studies have demonstrated that precise epigenomes reprogramming is required for this process, including the genome‐wide establishment, erasure, and reestablishment of epigenetic modifications.^22^, ^23^, ^24^ For instance, DNA methylation of parental genome is lost largely after fertilization.^25^, ^26^ Moreover, some histone modifications (like H3K4me3) exhibit noncanonical pattern which are inherited from oocytes then are established in the canonical pattern along with the embryo development.^23^ Besides these conventional epigenetic modifications, chromatin architecture and the RNA m^6^A modification are recently founded to be dynamically regulated during MZT.^27^, ^28^ In addition, epigenetic reprogramming defects can cause developmental defects and reproductive problems.^29^, ^30^, ^31^, ^32^, ^33^ Deciphering how and why this process occurs during early embryogenesis is crucial for addressing reproduction problems. However, constrained by scarcity of mammalian early embryos and technical limitations, global reprogramming of the epigenetic landscape during MZT and precise regulatory network of this complex process remain enigmatic for a long time. Recently, thanks to the advanced ultrasensitive chromatin analysis technologies, it is possible to measure the epigenome dynamics and illuminate the underlying molecular mechanisms during early embryo development. Here, we reviewed the current knowledge of epigenetic regulation in the MZT, focusing on DNA methylation, histone modification, chromatin structure, and RNA m^6^A modification. Furthermore, the post‐translational modifications of nonhistone proteins and candidate regulators participated in the MZT were also highlighted.

## 2 EPIGENETIC MODIFICATIONS

**FIGURE 2:**
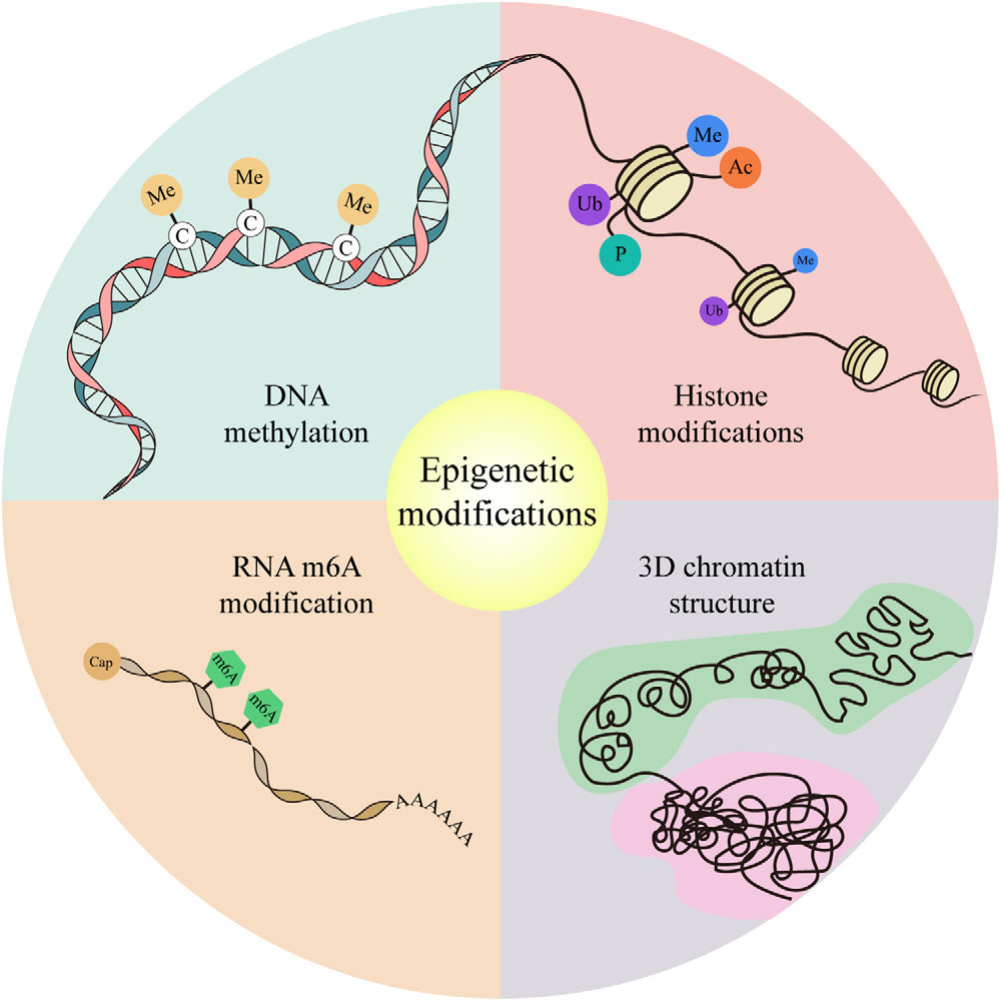
General schematic of epigenetic modifications in eukaryotes. In eukaryotes, DNA methylation is the well‐studied epigenetic modification and occurs at the 5′ position of the cytosine pyrimidine. DNA wraps around histone octamers, forming chromatin in nucleus. Histone modifications mainly consist of methylation, acetylation, ubiquitination, and phosphorylation. Moreover, chromatin is folded and organized three‐dimensionally within the nucleus. Besides, RNA epigenetic modification is intensely studied in recent years. RNA m6A is founded to be the most abundant mRNA modification.

Epigenetic modifications can cause heritable changes in gene expression but do not alter the nucleotide sequence.^34^, ^35^, ^36^, ^37^ DNA methylation is the most wildly studied epigenetic modification and occurs at the 5‐carbon position of cytosine residues (5mC) predominantly in CpG regions.^38^, ^39^, ^40^ Moreover, DNA methylation of enhancers and promoters is generally associated with the epigenetic silencing of transcription, by inhibiting the binding of activator proteins or recruiting repressor proteins to DNA.^41^, ^42^ In eukaryotes, DNA is organized to chromatin together with histone proteins.^43^, ^44^ Histone modifications can also directly impact on chromatin, alter the DNA‐templated processes, and affect gene regulation.^45^, ^46^ Different types of histone modifications cover distinct genomic regions and have diverse functions. Generally, H3K4me3 is associated with transcriptional initiation and occurs in the promoter region. H3K9me3 is constitutive hallmark of heterochromatin and associated with the silencing of repeat elements.^47^, ^48^ H3K27me3 in promoter regions is also highly correlated with gene repression.^49^ Moreover, H3K27ac is well recognized as a marker for active promoters and enhancers.^50^ In addition, the eukaryotic chromatin is not existed as a liner strand but folded and organized three‐dimensionally inside the nucleus.^51^ Chromatin 3D structures can intrinsically participate in gene regulation by influencing long‐range genomic interactions among regulatory elements and genes as well as the chromatin accessibility.^52^, ^53^ Besides these conventional modifications, RNA modification as a novel epigenetic modification has been intensely investigated in recent years.^54^, ^55^ RNA m6A, the most abundant mRNA modification, is reported to be associated with the mRNA stability, transport, and translation.^56^, ^57^ The main epigenetic modifications are summarized in Figure 2. With the recent advances in sequencing technology, we appreciate that global epigenetic reprogramming occurs during MZT and plays a crucial role in regulating transcription activity and transcript stability during this process (see below).

## 3 DNA METHYLATION IN MZT

DNA methylation is the most widely studied form of epigenetic modification and plays an important role in regulating gene expression.^58^ The proper reprogramming of DNA methylation is critical to the generation of embryos with totipotency.^59^ The genome in gamete is highly methylated, and the DNA methylation level of the paternal genome is originally considerably higher than that of the maternal genome.^60^, ^61^ After fertilization, extensive global DNA demethylation occurs across the genome, a process which is conserved among different mammals.^62^

### 3.1 Genome‐wide DNA demethylation after fertilization

**FIGURE 3:**
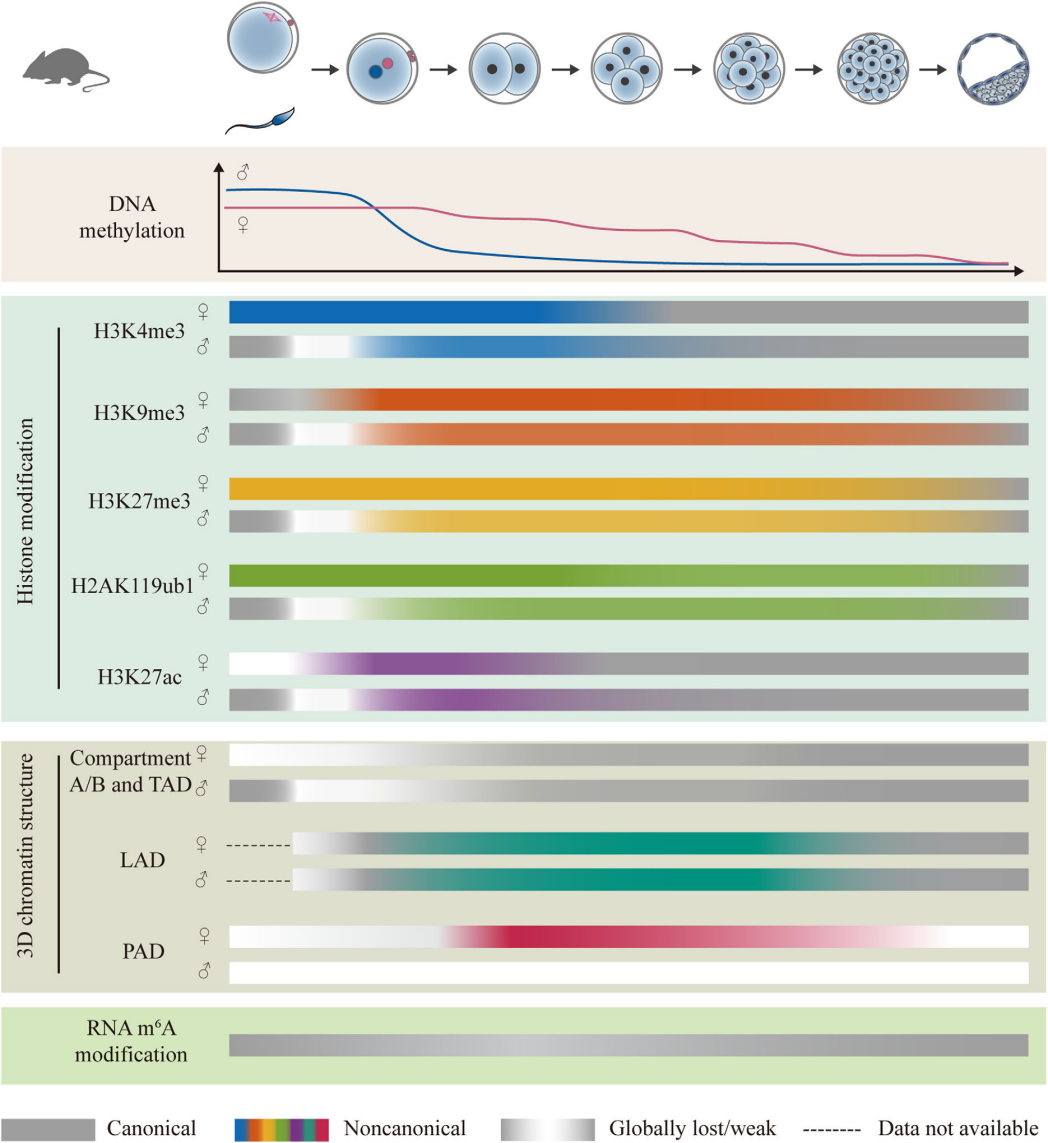
**Epigenetic reprogramming during early mouse embryo development**. Following fertilization, early embryos undergo global DNA demethylation. Maternal noncanonical H3K4me3, H3K27me3 (besides promoter H3K27me3), and H2AK119ub1 are inherited from oocytes, whereas paternal noncanonical histone modifications and maternal noncanonical H3K27ac are de novo established after fertilization. H3K4me3 and H3K27ac are reprogrammed to the canonical patterns at the 2‐cell stage. Noncanonical H3K9me3, H3K27me3, and H2AK119ub1 remain until the blastocyst stage. Compartments A/B and topologically associating domains (TADs) are largely lost after fertilization and become consolidated gradually with the embryo development. Polycomb‐associating domains (PADs) can only be detected in the maternal genome in early embryos and become clear at the 2‐cell stage but absent in blastocysts. Lamina‐associated domains (LADs) exist as a noncanonical pattern on both parental genomes at the 2‐cell stage and reprogrammed to canonical pattern in 8‐cell embryos. Global abundance of m^6^A decreases continuously during mouse maternal‐to‐zygotic transition (MZT) but increases after the 2‐cell stage.

DNA methylation in oocytes is distinct, covering approximately 40% of the genome, and is exclusively deposited in actively transcribed gene bodies. Furthermore, DNA methylation is evenly distributed and covers about 90% of the sperm genome.^63^, ^64^, ^65^ Following fertilization, early embryos undergo global DNA demethylation (besides imprinting control regions (ICRs) and certain repetitive sequences) mediated by active and passive mechanisms, which are conserved between mice and humans (Figure 3).^62^ Generally, the paternal genome is rapidly demethylated shortly after fertilization (<12 h), whereas the maternal genome demethylated gradually after several cell divisions in both mouse and human preimplantation embryos.^61^, ^66^ Both parental genomes undergo passive and active demethylation, and active demethylation in the paternal pronuclei is more robust than that in the maternal pronuclei.^66^, ^67^, ^68^ Passive demethylation is through replication‐dependent dilution when maintenance‐type DNA methyltransferase DNMT1 is unlocalized in the nucleus or unfunctional.^58^ Yan et al. found that maternal NLRPL4 may be required for the cellular localization of DNMT1 and its cofactor UHRF1.^69^ By immunostaining, they found that DNMT1/UHRF1 complex is localized not only in the cytoplasm but also in the pronuclei of the *Nlrp14* maternal knockout (maternal KO) zygotes.^69^ NLRPL4 deficiency impairs DNA demethylation in zygotes and results in developmental arrest at the 2‐cell stage. Further, the treatment of DNMT inhibitor 5‐Aza could reduce 5mC level in the KO embryos.^69^ For active DNA demethylation, a well‐known mechanism is that TET3 catalyzes the oxidation of 5mC to 5hmC, 5fC, and 5caC on the genome, but it remains unclear how modified cytosines finally replaced by unmodified ones.^58^ In mouse embryonic stem cells (mESCs), thymine‐DNA glycosylase (TDG)‐mediated base excision repair is essential for TET3‐mediated active demethylation by excising the oxidized forms of 5mC, including 5fC and 5caC.^70^ However, TDG maternal deficiency does not affect the immunostaining signals of 5mC, 5hmC, and 5caC in mouse zygotes.^68^ Moreover, the majority of oxidized 5mC sites are converted to cytosines independent of the passive dilution.^71^ These results indicated the existence of TDG‐independent and TET3‐mediated active demethylation mechanisms in early embryos.^71^, ^72^

### 3.2 Controversial mechanisms of DNA demethylation in pronuclei

The maternal genome was demethylated mainly in a passive manner rather than active manner.^66^, ^73^ Although an increased level of 5hmC can be also detected in the maternal pronuclei, it is much milder than that in the paternal pronuclei.^68^, ^69^ This difference may attribute to the asymmetric H3K9 methylation in the maternal pronuclei which is thought to protect the maternal genome from TET3 oxidation.^74^, ^75^ One possible mechanism is that the intranuclear STELLA can inhibit the enzymatic activity of TET3 on the genome through binding to H3K9me2‐containing chromatin.^75^, ^76^ Overexpression of H3K9me/H3K9me2‐specific demethylase JHDM2A abolished H3K9me2 that results in STELLA removal, 5mC reduction, and 5hmC elevation in maternal pronuclei of mouse zygotes.^75^ Furthermore, STELLA maternal deficiency or driving STELLA out of the nucleus by RanBP5‐mER expression also induces the conversion of 5mC to 5hmC in the maternal pronuclei.^75^, ^76^

However, the predominant DNA demethylation in paternal pronuclei remains controversial. The classical model postulates that the paternal genome undergoes extensive DNA demethylation before replication and maternally inherited TET3 plays an important role in this process, which was based on the results of immunofluorescence staining.^77^, ^78^, ^79^ However, using the technique of reduced representation bisulfite sequencing, some groups found that DNA replication is the major contributor to paternal genome demethylation because the majority of demethylated CpG sites depend on DNA replication rather than TET3 in the paternal genome in mouse zygotes.^67^, ^68^ Interestingly, another group reached the opposite conclusion using the bisulfite sequencing technology that the global demethylation mainly proceeds through active demethylation manner.^71^ However, Amouroux et al. found that the inhibition of DNA replication does not affect the loss of 5mC and the generation of 5hmC in paternal pronuclei through ultrasensitive liquid chromatography–mass spectrometry.^80^ It should be noted that bisulfite conversion cannot distinguish 5mC from 5hmC; thus, the results could be affected by the replication‐induced dilution.^81^ Given that DNMT3A and DNMT1 are reported to be required for TET3‐mediated 5hmC formation, and the level of 5hmC is significantly higher in demethylated loci rather than other loci in the zygote, TET3‐mediated active DNA demethylation may play an important role in protecting the newly formed hypomethylated regions from re‐methylation.^69^, ^80^ But the normal preimplantation development of reconstructed zygotes that bypass paternal 5mC oxidation notes the TET3‐mediated 5mC oxidation on the paternal genome is dispensable for mouse embryo development.^82^ The deficiency of maternal TET3 causes mouse neonatal sub‐lethality, but it is attributed to the haploinsufficiency at the neonatal stage rather than the defective paternal 5mC oxidation in the zygotes.^77^, ^82^ Indeed, the moderate impact to DNA demethylation and early mouse embryo development induced by TET3 loss also implies the existence of undiscovered redundant TET3‐independent active demethylation in early embryos.^67^, ^80^

### 3.3 Maintenance and de novo DNA methylation after fertilization

In fact, DNA demethylation is accompanied by selective maintenance and de novo methylation. Some genomic regions, including ICRs and certain repetitive sequences, remain methylated during global demethylation in early embryos.^72^ In mice, besides the abovementioned STELLA which can protect the maternal genome from TET3‐mediated demethylation, KRAB zinc finger protein ZFP57 is also reported to selectively recognize and bind to methylated ICRs and recruit DNA methyltransferases via KAP1/TRIM28 to maintain both maternal and paternal imprints.^75^, ^76^, ^83^ In human, another member of KRAB zinc finger protein ZNP445 plays a predominant role in imprinting maintenance.^84^ Moreover, using isotope labeling, Amouroux et al. found the presence of de novo DNA methylation activity in the late mouse zygotes.^80^ In human, de novo methylation occurs with two waves, one is at the 1‐cell stage on the paternal genome and the other is from the 4‐ to 8‐cell stage.^61^ Moreover, the de novo methylation occurs in regions enriched for repeat elements, likely repressing activation of these regions.^61^ Further studies are warranted to elucidate the exact mechanisms supporting DNA methylation reprogramming and reveal their importance for embryo development.

## 4 HISTONES MODIFICATIONS IN MZT

**FIGURE 4:**
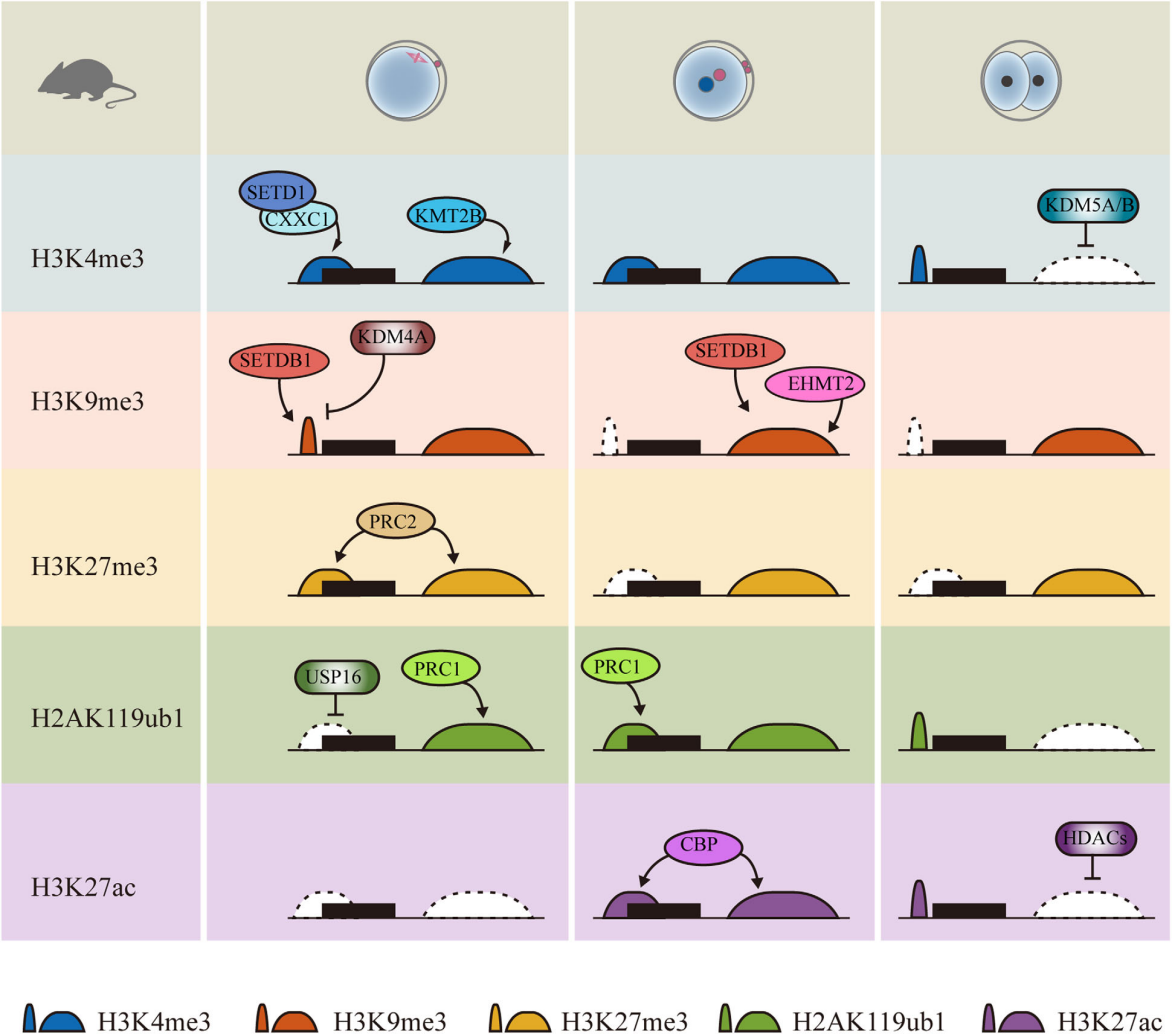
**Dynamic changes and regulations of histone modifications on the maternal genome during maternal‐to‐zygotic transition (MZT)**. In MII oocytes, ncH3K4me3 is deposited in both promoters and distal regions, which was mediated through the action of KMT2B and SETD1‐CXXC1. Moreover, ncH3K4me3 is removed by KMT2A/B in 2‐cell stage embryos, facilitating the formation of canonical H3K4me3. Correct distribution of H3K9me3 in oocytes requires SETDB1 and KDM4A. The H3K9me3 at promoter regions is largely removed after fertilization. In zygotes, the deposition of H3K9me3 on maternal genome is mediated by SETDB1 and EHMT2. Both distal domains and promoters are marked by H3K27me3 in MII oocytes. H3K27me3 at promoter of developmental genes is removed after fertilization, but H3K27me3 at distal regions are inherited by the zygote. USP16 and PRC1 ensure the normal distribution of H2AK119ub1 in oocytes. Moreover, PRC1 guides the reestablishment of H2AK119ub after fertilization, occupying both promoter regions and distal regions. Moreover, the H2AK119ub1 is removed from broad distal domains and is deposited in promoter regions. The ncH3K27ac is established after fertilization, and CBP contributes to this process. In 2‐cell stage embryos, HDACs are responsible for the transition of broad H3K27ac domains to narrow peaks.

Histones are the main protein components of chromatin, and their post‐translational modifications play pivotal roles in the regulation of chromatin structure remodeling and many biological processes.^85^ Various studies have demonstrated that the proper reprogramming of histone modification is essential for early embryo development. Moreover, using advanced low‐input ChIP‐seq and CUT&RUN techniques, genome‐wide profiling of histone modification can be generated in early embryos. Interestingly, some histone modifications represent noncanonical patterns in oocytes and pre‐implantation embryos. Recent findings on dynamics and underlying mechanisms of histone modification reprogramming during MZT are summarized as follows (Figures 3 and 4).

### 4.1 H3K4me3

#### 4.1.1 Dynamic changes in H3K4me3 levels and status during MZT

H3K4me3 is strongly enriched in promoter region of active genes in nongrowing oocytes (NGOs), whereas it exists as a noncanonical pattern (ncH3K4me3) that covers broad domains in promoters, gene bodies, and intergenic regions in mouse oocytes at subsequent stages.^86^, ^87^, ^88^ Moreover, the majority of distal ncH3K4me3 marked regions are overlapped with oocyte partially methylated domains (PMDs).^87^ After fertilization, the oocyte ncH3K4me3 is inherited by zygotes and maintained until the 2‐cell stage.^86^, ^87^ By contrast, the sperm H3K4me3 is rapidly erased after fertilization and replaced by a low level of very broad H3K4me3.^87^ Upon the onset of the major ZGA, the ncH3K4me3 is removed, with the canonical H3K4me3 restricted to active promoters starting to be established.^87^

However, H3K4me3 is enriched in promoter regions in human germinal vesicle (GV) and MI oocytes, unlike the broad ncH3K4me3 in mouse oocytes. Moreover, in human 4‐cell embryos, the H3K4me3 of promoter is wider than that at other stages and about half of these promoters are activated upon ZGA.^89^ The widespread distal H3K4me3 also appears in CpG‐rich and hypomethylated regions in human 4‐cell embryos but whether its function is conserved between mice and humans warrants further investigation.^89^

#### 4.1.2 Formation of H3K4me3 during oogenesis

Zygote ncH3K4me3 is inherited from oocytes. It is reported that KMT2B and CXXC1‐SETD1 are required for the deposition of H3K4me3 on oocyte genome, functioning complementarily by targeting different areas of the oocyte genome.^90^ H3K4 histone methyltransferase KMT2B (also known as MLL2) is responsible for the establishment of ncH3K4me3 domains in mouse oocytes.^86^, ^88^ Oocyte‐specific depletion of *Kmt2b* with *Gdf9‐Cre* leads to oocyte loss, abnormal ovulation, and female sterility, accompanied by significantly decreased H3K4me3.^91^ Importantly, KMT2B is also important for early embryo development, especially for the first cell fate decision. The maternal deficiency of *Kmt2b* causes the arrest of majority of embryos between one‐ and four‐cell stages with defective ZGA, and the dramatic decrease of H3K4me3 in these embryos.^91^ ChIP‐seq analysis reveals that H3K4me3 profile of *Kmt2b* KO oocytes is similar to that of WT NGO oocytes, lacking the broad domains and distal peaks, which further indicates that *Mll2* is required for the deposition of ncH3K4me3 but not canonical H3K4me3 during the process of oogenesis.^88^ H3K4me3 in mouse NGOs enriched in the active promoters marked with H3K27ac and associated with transcription activation. As oogenesis developed, the deposition of ncH3K4me3 increases largely in mouse fully grown GV oocytes (FGO) in a transcription‐independent manner, targeting intergenic regions, silent promoters marked with H3K27me3 (bivalent domains), and distal genomic elements, such as putative enhancers marked with H3K27ac.^88^ Interestingly, the deletion of *Kmt2b* has a limited influence on gene expression in GV oocytes but causes defective transcriptional repression in peri‐ovulatory oocytes.^88^, ^91^ It is possible that the maternal ncH3K4me3 existed in oocytes and early embryos executes transcriptional repressive function.^88^, ^91^ One possibility is that the distal ncH3K4me3 may work as “sponges” to attract transcriptional resources, such as TAF3, therefore preventing premature transcription before ZGA, but also preparing for the rapid activation after ncH3K4me3 is removed in zygotes.^87^, ^88^, ^92^

It was reported that the SETD1 histone H3K4 methyltransferase complex contributes to the generation of H3K4me3 in oocytes.^90^ Depletion of *Setd1a* in oocytes has no impact on fertility.^93^ However, *Setd1b* oocyte‐specific knockout reduces the amount of follicular. The ovulated MII oocytes exhibit an abnormal distribution of cytoplasmic organelles, accumulated lipid droplets, and dysregulated gene expression. Consequently, most *Setd1b* null embryos are arrested at the pronuclear stages with defective zona pellucida.^94^ Although the global abundance is normal, the redistribution of H3K4me3 occurs in *Setd1b* null oocytes.^94^, ^95^ In detail, H3K4me3 is lost in active gene promoters which associated with the alterations in gene expression after *Setdb1* deletion. Meanwhile, H3K4me3 is widely deposited in MLL2 targeted regions that are across CpG‐rich sequences and hypomethylated DNA, indicating increased or misdirected KMT2B activity in certain regions.^95^

CXXC finger protein‐1 (CXXC1, also known as CFP1) is the key subunit of the SETD1 component, which can recognize and bind to specific genomic regions. Similar to SETDB1, maternal CXXC1 is essential for oocyte maturation and embryo developmental after fertilization.^96^, ^97^ In contrast, the depletion of CXXC1 causes a marked decrease of H3K4me3 level, regardless of canonical and noncanonical peaks.^96^, ^97^, ^98^, ^99^, ^100^ ChIP‐Seq results further indicated that CXXC1 is essential for the establishment of H3K4me3 in promoter regions modified by H3K4me3 exclusively, whereas KMT2B is required for regions also occupied by H3K27me3.^96^ Correspondingly, global transcription activity is downregulated after *Cxxc1*‐maternal deletion, including the genes correlated with mRNA synthesis, translation, and degradation.^98^, ^100^ Particularly, impaired translation of transcripts encoding maternal factors (like *Btg4*) important for maternal mRNA decay further prevented the maternal mRNAs degradation in mouse CXXC1 null oocytes and zygotes.^98^, ^100^

#### 4.1.3 Global demethylation of H3K4me3 at the 2‐cell stage embryos

In late 2‐cell stage embryos, ncH3K4me3 is replaced by canonical H3K4me3, and ZGA is indispensable for this transition.^87^ Upon the treatment of embryos with α‐amanitin before major ZGA, the removal of ncH3K4me3 cannot be observed at the 2‐cell stage embryos. H3K4 demethylases KDM5A/5B may be required for the active removal of ncH3K4me3 in mouse early embryos. Therefore, the knockdown of *Kdm5a/5b* leads to a high level of H3K4m3 at the late 2‐cell stage, as well as the downregulation of a large number of ZGA genes and developmental arrest before the blastocyst stage.^86^

#### 4.1.4 Relationship between ncH3K4me3 and DNA methylation

In oocytes, H3K4me3 and DNA methylation are mostly mutually exclusive on the genome, and the deposition of these two modifications occurs in parallel.^88^, ^95^ The mechanisms underlying this phenomenon are bidirectional. It is found that DNA methylation can repel KMT2B and inhibit the H3K4me3 accumulation in methylated regions.^101^ Consistently, DNA methylation is mostly deposited in the gene body region of transcribed genes in oocytes.^60^, ^65^ When DNA methylation is absent, H3K4me3 is evidently enriched in gene bodies that are normally methylated in GV oocytes.^88^ Conversely, the preexisting H3K4me3 can repel DNA methyltransferases to inhibit DNA methylation.^102^, ^103^, ^104^ Some studies found that DNA methylation is decreased in either *SETD1B* or *CXXC1* deficiency mouse oocytes, accompanied by the unchanged abundance of DNMTs.^95^, ^96^ However, it is still unclear whether an abnormal accumulation of H3K4me3 blocks the de novo DNMTs.^95^ Owing to that H3K4me3 is highly enriched in normally DNA methylated regions, it is also possible that the delayed DNA methylation caused by SETD1B deletion created an environment for MLL2‐mediated H3K4me3 deposition.^95^ Moreover, loss of ncH3K4me3 caused by the deletion of KMT2B has a limited impact on DNA methylation in mouse oocytes.^88^ The precise interaction between DNA methylation and H3K4me3 formation still needs further in deep investigation.

### 4.2 H3K27me3 and H2AK119ub1

#### 4.2.1 Dynamic changes in levels and status of H3K27me3 and H2AK119ub1 during MZT

H3K27me3 and H2AK119ub1, two hall markers of polycomb domains, are reprogrammed during early embryo development.^105^ In mouse oocytes, distributions of both the H3K27me3 and H2AK119ub1 are noncanonical with the weak enrichment in promoter regions and board domains in distal regions, particularly the PMDs.^106^ Moreover, a significant overlap between H2AK119ub1 and H3K27me3 can be seen in both oocytes and sperms. The colocalization is largely lost during preimplantation development and regained in a canonical form after implantation.^106^

After fertilization, H3K27me3 in promoter regions of developmental‐related genes is largely erased in mouse embryos and reestablished in post‐implantation embryos.^107^ By contrast, noncanonical H3K27me3 in distal regions inherited from oocytes is present throughout preimplantation development.^106^, ^108^ In the paternal pronuclei, sperm H3K27me3 is erased upon fertilization and a broad H3K27me3 domain in gene desert regions appears in the zygotes.^107^ In human oocytes, H3K27me3 is also enriched in both promoters and PMDs. However, unlike that in mouse embryos, H3K27me3 is widely erased after fertilization and maternal‐specific H3K27me3 are retained at the 2‐cell stage embryos but nearly absent at the 8‐cell stage in human embryos (unlikely act as an imprinting marker).^89^, ^109^

For H2AK119ub1, some groups found that the newly established H2AK119ub1 in paternal pronuclei is mainly enriched in gene desert regions in zygotes. In maternal pronuclei, the distribution of H2AK119ub1 on maternal genome largely resembles that in MII oocytes, which indicated the inheritance from oocytes. Sperm H2AK119ub1 is largely erased after fertilization. Moreover, the H2AK119ub1 domains on maternal genome are more than that on paternal genome in zygotes.^106^, ^110^ H2AK119ub1 is removed in broad distal domains and progressively deposited at typical polycomb targets canonically after 1‐cell stage, leading to a parental comparable distribution at the late 2‐cell stage embryos.^106^, ^110^, ^111^ Controversially, a recent study indicated that the H2AK119ub1 is largely diminished after meiotic resumption and nearly undetectable in MII oocytes, arguing that the maternal H2AK119ub1 in zygotes is newly established after fertilization.^112^ Notably, the dynamic changes in levels and status of H3K27me3 and H2AK119ub1 during MZT might be critical for early embryo development.

#### 4.2.2 Formation of H3K27me3 and H2AK119ub1 during oogenesis

The establishment of H3K27me3 is catalyzed by PRC2 during mouse oocyte growth.^113^ Evidences indicate that oocyte H3K27me3 seems to be unnecessary for oogenesis and early embryogenesis. PRC2 deficiency in oocytes causes the loss of H3K27me3 but does not affect oocyte growth and maturation, even the preimplantation development of embryos derived from these oocytes.^114^, ^115^ In addition, PRC2 is absent in human oocytes and pre‐ZGA embryos, which indicated that human H3K27me3 is established at an earlier embryonic stage.^89^, ^109^

H2AK119ub1 is co‐established with H3K27me3 during oocyte growth, which is mediated by PRC1 and participates in transcriptional repression.^110^, ^116^ Oocyte‐specific deletion of RING1A/1B, two core components of PRC1 components, causes absent of H2AK119ub1, aberrant transcription in oocytes, impaired ZGA, and developmental arrest at the 2‐cell stage.^117^ After meiotic resumption, Rong et al. found that the level of H2AK119ub1 is promptly declined genome‐widely, and USP16 is indicated to be the major contributor to this meiotic‐coupled H2AK119ub1 deubiquitination.^112^ Other deubiquitinases, like USP21, also possess H2AK119 deubiquitinating activity but fails to support their function normally due to the low abundance. Although it has a mild impact on oocyte generation and maturation, USP16‐mediated H2AK119ub1 removal during meiosis is indispensable for the proper MZT. Maternal *USP16* deletion prevents the ZGA and causes nearly half of the embryos arrested at the 2‐cell stage, which may be attributed to the retention of H2AK119ub1 at the TSSs of early zygotic genes in oocytes. It seems that the removal of maternal H2AK119ub1 regulated by USP16 may be a prerequisite for proper ZGA at the 2‐cell stage embryos.^112^

Interestingly, there are some interactions between the formations of H3K27me3 and H2AK119ub1. The removal of H3K27me3 upon *PRC2* depletion does not affect H2AK119ub1 in oocytes but disrupts the deposition of H2AK119ub1 at some H3K27me3 imprinting loci in morulae.^106^ The *PRC1* deficiency not only severely impairs the formation of H2AK119ub1 but also causes gene‐selective loss of H3K27me3 in FGOs. More importantly, this H3K27me3 loss is inherited by embryos, leading to defective H3K27me3‐dependent imprinting and placental enlargement.^110^ However, the acute removal of H2AK119ub1 in zygotes has no impact on H3K27me3 imprinting in early embryos.^106^ Understanding the underlying mechanisms for the detailed correlation between H2AK119ub1 and H3K27me3 in early development embryos will benefit the exploration of some epigenetic mechanisms for the abnormal development of early embryos.

#### 4.2.3 Reprogramming of H3K27me3 and H2AK119ub1 after fertilization

After fertilization, the H3K27me3 on promoter is largely erased in a ZGA‐independent (minor or major) manner.^107^ Moreover, the maintained maternal noncanonical H3K27me3 inherited from oocytes controls DNA methylation‐independent imprinting throughout preimplantation stages of mouse embryonic development.^106^, ^108^ H3K27me3‐specific demethylase *Kdm6b* mRNA injection affects the paternal allele‐biased expression of putative H3K27me3‐dependent imprinted genes rather than the canonical imprinted genes.^118^ Although H2AK119ub1 and H3K27me3 are colocalized in gametes, the resetting of H2AK119ub1 is decoupled with H3K27me3 after fertilization. The H2AK119ub1 level is dramatically increased at 2‐cell stage embryos and maintained at a high level at the subsequent stages. Consistently, RNF2, the core component of PRC1, is highly expressed and localized in the nucleus of early embryos.^112^ Recent studies indicated that H2AK119ub1 retained in developmental genes promoter regions after fertilization may play a predominant role in preventing premature activation of these genes during ZGA and early developmental embryos.^106^ However, the maternal bias of H2AK119ub1 at H3K27me3‐dependent imprinting loci is nearly absent during preimplantation development, which indicated the dispensable role of H2AK119ub1 on maintenance of noncanonical imprinting.^106^, ^111^

#### 4.2.4 Relationship among H3K27me3, H2AK119ub1, and H3K4me3

H2AK119ub1 mostly deposited in the promoters of transcriptionally silenced genes that modified by H3K27me3.^110^, ^112^ Besides colocalized with H3K27me3, H2AK119ub1 is also found to be preferentially enriched in the promoter of active transcribed genes marked by H3K4me3 in mouse GV oocytes.^87^, ^110^, ^112^ Although genes with H2AK119ub1/H3K4me3 at promoters are actively transcribed, the expression levels are relatively lower than those with H3K4me3 only.^112^ Moreover, among these genes with promoter H2AK119ub1/H3K4me3, the upregulated genes are much more than the downregulated genes after H2AK119ub1 ablation in *Ring1a/1b* knockout mouse FGOs.^110^ Generally, H2AK119ub1 at promoters seems to play a role in repress the transcriptional activity when coexisting with other histone.^87^ Moreover, the inhibitory effect of H2AK119ub1 should be weaker than H3K27me3.^58^, ^96^ H3K27me3 and H2K4me3 were also observed to be colocalized at bivalent promoters of developmental genes. Moreover, the genes modified by H3K27me3 in promoter regions are silenced regardless of the deposition of other two modifications.^96^ How the three modifications affect each other could be more clearly illustrated by the manipulation of their related genes in mouse models that can easily be achieved by the CRISPR/Cas9‐mediated gene editing technology.

### 4.3 H3K9me3

#### 4.3.1 Dynamic changes of H3K9me3 during MZT

After fertilization, the parental H3K9me3 undergoes global reprogramming in mouse embryos.^119^ The majority of sperm H3K9me3 and half of oocyte H3K9me3 domains are quickly lost after fertilization. In addition, both parental H3K9me3 undergo widely de novo establishment in zygotes, and H3K9me3 domains on maternal genome are significantly more than that on paternal genome.^119^ H3K9me3 domains in promoter regions are depleted after fertilization and reestablished at the post‐implantation stage.^119^ Unlike the loss of H3K9me3 in promoter regions, the number of long terminal repeats (LTRs) marked by H3K9me3 increases gradually.^119^

#### 4.3.2 Formation of H3K9me3 during oogenesis

In oocytes, SETDB1 mediates the deposition of H3K9me2/3 and is essential for meiotic progression and preimplantation development.^120^ Besides, H3K9me3 demethylase KDM4A mediates the removal of H3K9me3 at broad H3K4me3 domains in mouse oocytes, which is crucial for the proper activation of genes and transposable elements during ZGA.^121^ It is observed that the aberrant enrichment of H3K9me3 in regions normally occupied H3K4me3 in *KDM4A* ablation oocytes. Moreover, embryos derived from these oocytes show defective ZGA and preimplantation lethality.^121^ This finding echoes the reports in SCNT embryos that incomplete depletion of H3K9me3 is a major epigenetic barrier, and excessive H3K9me3 prevents topologically associating domains (TADs) removal and induces ZGA failure. However, the defects can be rescued by the overexpression of H3K9me3 demethylase KDM4B/4D or by the deletion of H3K9 methyltransferase *SUV39H2*.^122^, ^123^, ^124^

#### 4.3.3 Reprogramming of H3K9me3 after fertilization

In zygotes, the bias deposition of H3K9 methylation in the maternal pronuclei contributes to preventing TET‐mediated 5mC oxidation on maternal genome, which is mediated by EHMT2 and SETDB1.^125^ De novo establishment of H3K9me3 in the paternal pronuclei after fertilization is catalyzed by SUV39H2 and may contribute to subsequent establishment of heterochromatin in mouse embryos.^126^ Importantly, the H3K9me3 plays an important role in the transcriptional repression of LTRs after the 2‐cell stage. After fertilization, a large number of LTRs, including MERVL, are highly expressed, possibly because of the global demethylation and other relief of epigenetic repression. The subsequent repression of LTRs requires the establishment of H3K9me3, and CHAF1A is essential for H3K9me3‐mediated LTR silencing.^47^, ^119^ In human, oocyte‐specific H3K9me3 is quickly eliminated after fertilization and is mainly enriched in LTRs until the 8‐cell stage. Therefore, similar with that in mouse embryos, H3K9me3 also plays a critical role in LTRs silencing in human embryos.^127^

### 4.4 H3K27ac

#### 4.4.1 Dynamic changes in levels and status of H3K27ac during MZT

H3K27ac exists as a noncanonical broad pattern (ncH3K27ac) in GV stage and becomes very weak in MII mouse oocytes.^128^ In mouse zygotes, high level of ncH3K27ac is reestablished precedes minor ZGA. Particularly, an establishment of ncH3K27ac on paternal genome is earlier than that on maternal genome. Moreover, paternal ncH3K27ac exhibits higher level and broader domains than maternal ones.^128^ Global H3K27ac level is reduced at the 2‐cell stage embryos with the erasure of ncH3K27ac and de novo establishment of canonical narrow H3K27ac.^128^, ^129^ Notably, the immunostaining signal of H3K27ac progressively increases after the 4‐cell stage.^129^ Similarly, in human, the immunostaining signal of H3K27ac is high in the zygotes and 2‐cell embryos and reduced in 8‐cell embryos (the timing of human major ZGA).^128^ Recently, Wu et al. further indicated that genome‐wildly distributed broad H3K27ac domains are also observed in 2‐ and 4‐cell human embryos and turn into narrow and typical peaks in 8‐cell human embryos.^130^

#### 4.4.2 Reprogramming of H3K27ac after fertilization

Broad ncH3K27ac domains are mainly located in gene dense regions and strongly correlated with ncH3K4me3 in mouse zygotes.^128^ The acetyltransferase CBP/p300 is required for the broad deposition of ncH3K27ac. Inhibition of CBP/p300 with inhibitor A‐485 leads to the loss of H3K27ac and the developmental arrest at the 2‐cell stage.^128^ Furthermore, minor ZGA defect is detected in CBP/p300‐inhibited embryos, and H3K27ac is enriched in promoters of minor ZGA genes coinciding with chromatin opening rather than major ZGA genes, which indicated that ncH3K27ac may contribute to the activation of minor ZGA genes.^128^, ^129^ Moreover, the level of H3K27ac is decreased in 2‐cell embryos. Both HDACs and NAD^+^‐SIRT1‐mediated deacetylation facilitate the erasure of H3K27ac and the timely silencing of minor ZGA genes.^128^, ^129^ Indeed, TSS regions of major ZGA genes are pre‐configurated with H3K27ac in early 2‐cell embryos that may provide a permissive state for major ZGA. Moreover, H3K27ac domains in distal regions dependent on CPB/p300 induces distal opening elements that may function as enhancers for major ZGA.^128^

In human, broad H3K27ac domains are highly correlated with broad H3K4me3 domains before ZGA. Moreover, HDACs are required for the transition of broad H3K27ac domains to narrow peaks at the ZGA stage.^130^ Interestingly, the NAD^+^ supplement promotes the removal of H3K27ac and improves the blastocyst rate of human intracytoplasmic sperm injection (ICSI) embryos. It seems that the NAD^+^‐SIRT1‐mediated deacetylation also plays an important role in human embryo development.^129^

## 5 3D CHROMATIN STRUCTURE IN MZT

In eukaryotic cells, the genomic DNA is packaged into a hierarchical structure in nucleus, which is essential for proper regulation of gene expression.^131^ Using low‐impute chromosome‐conformation‐capture technologies, recent studies have profiled the dynamics of the 3D chromatin structure during early embryo development.^27^ However, the role of the higher order chromatin structure in early embryo development is largely unknown. Here, we summarized the latest findings on the dynamics of 3D chromatin structure in mammalian embryos during MZT (Figure 3).

### 5.1 Compartment A/B

Studies using Hi‐C technology have revealed that the entire genome can be divided into two compartments at a solution of 1Mb, called compartment A/B.^132^ Compartment A is associated with open and active chromatin, and compartment B is densely packed and related with closed chromatin.^132^ Compartments are found in mouse FGO but absent in MII oocytes.^133^, ^134^, ^135^ In addition, sperm can be partitioned into A/B compartments.^136^ After fertilization, an extensive loss of compartments A/B occurs in both parental pronuclei, with maternal genome showing relatively weaker compartments than paternal genome in mouse zygotes.^134^ Compartment segregation is clear at the 2‐cell stage and consistent until the late stage in mouse embryos.^134^ In human embryos, A/B compartmentalization is lost at the 2‐cell stage and is very weak at the 8‐cell stage but is clear at the morula stage.^137^ It has been reported that heterochromatin protein HP1 and H3K9me3 might be required for the establishment of compartment B in Drosophila early embryos.^138^ Therefore, to address the important role of compartment A/B in early embryo development, in deep investigation is warrant to explore whether the regulation of compartment A/B is conserved across different species.

### 5.2 TADs

TADs are smaller units of 3D nuclear organization with two features: self‐association and insulation from regions outside the domains.^139^ In mice, similar to compartment A/B, TADs are also present in FGO oocytes but absent in matured MII oocytes.^133^, ^134^, ^135^ Moreover, TADs are also exhibited in mouse mature sperms.^136^ However, human sperms do not have TADs.^137^ Whether TADs exist in human oocytes remains unknown. TADs are largely disassembled after fertilization and then slowly de novo established in mouse embryos.^134^ In mouse embryos, TADs are in priming state showing weak intra‐TAD interactions and boundary insulation at the zygote stage and become mature characterized by strong intra‐TAD until the 8‐cell stage.^134^ In human embryos, TADs are de novo established after fertilization and mature TADs can be detected at the morula stage.^137^ The formation of TADs requires cohesion and CCCTC‐binging factor (CTCF).^139^, ^140^, ^141^ Cohesion can extrude a chromatin loop with a ring‐shaped structure, and CTCF functions as anchor to block the cohesion motor. It has been indicated that TADs and DNA loops can mediate the long‐range chromatin interaction between the enhancer and the promoter to regulate gene expression.^142^, ^143^ Removal of cohesion leads to the TADs elimination in mouse early embryos.^144^ Likewise, it is found that TAD organization requires the expression of CTCF in human embryos.^137^ Additionally, chromatin remodeling factor ISWI is essential for TADs establishment in xenopus early embryos, possibly by mediating the CTCF binding on chromatin.^145^ Whether chromatin remodeling complex plays an essential role in establishing TAD structures in mammals needs further in deep investigation. Besides, TADs consolidation is reported to be independent of zygotic transcription in mouse embryos, whereas it is dependent on zygotic transcription in human embryos.^134^, ^137^ The difference between mouse and human may be due to the low level of architectural proteins related to TADs (such as CTCF) in human oocytes.^137^ The features with weak compartmentalization and priming TADs indicate that chromatin is likely to be in a relatively relaxed state after fertilization. Such relaxed state of chromatin as well as the step‐wise establishment of 3D genome may be required for resetting the memories coded in the chromatin structure in gametes.^27^ The molecular basis and the function for the reprogramming of chromatin structure in mammals remain to be explored.

### 5.3 LADs

Lamina‐associated domains (LADs) are genomic regions that interact with the nuclear lamina (NL), which are associated with the chromosome organize inside the nucleus and gene repression.^146^ Like other structures, LADs are also absent in mouse oocytes.^147^ After fertilization, LADs are de novo established independently of DNA replication and are clearly detected in mouse zygotes. In mouse zygotes, the paternal LADs are more typical, whereas maternal LADs are atypical with more fragmented patterns, fewer genome‐NL contacts and even increased DNaseI hypersensitivity.^147^ In 2‐cell embryos, LADs are reorganized to the atypical pattern, with the typical LADs dislodged from the NL and replaced by the intermediate LADs. Moreover, compared with other stages, a considerable part of LADs is overlapped with A compartments at the 2‐cell stage. The LADs display more typical features and lose parental differences in 8‐cell embryos. Intriguingly, it is proposed that de novo establishment of paternal LADs in zygotes may be dependent upon remodeling of H3K4 methylation.^147^ LADs in human gametes and embryos are largely unknown.

### 5.4 PADs

Domains that are self‐interacting, marked by noncanonical H3K27me3 and cohesion‐independent, are termed polycomb‐associating domains (PADs).^116^, ^148^ In mice, PADs are only present in early stage of oocytes.^116^ Maternal PADs can be detected in early 2‐cell embryos, become further clear in late 2‐cell embryos, and disappear in blastocysts. However, paternal PADs are absent in early mouse embryos.^116^ It was found that PRC1 but not PRC2 is indispensable for the establishment of PADs in mouse oocytes. In line with the observation in oocytes, PRC1 or H2AK119 is found to be responsible for the polycomb‐dependent chromosome interactions in mESCs.^140^ However, the reestablishment of PADs in mouse embryos requires PRC2 or H3K27me3, which is different from that found in oocytes.^116^ Further work is required to fully appreciate the role of PRC1/H2AK119ub and PRC2/H3K27me3 in regulating PADs. Notably, in addition to the attenuated PADs, genes in PADs are dramatically depressed in PRC1 absent mouse oocytes.^140^ It is speculated that PRC1‐mediated PADs may function to proper gene repression through separating the genome into defined compartments. Several published observations have also demonstrated that PRC1/H2AK119ub‐mediated polycomb chromatin domain formation and long‐range chromatin interactions are contributed to transcriptional repression in mESCs.^149^, ^150^, ^151^, ^152^ Besides, whether PADs play any role in ZGA and embryo development remains unclear. Moreover, the roles of PADs in human gametes and embryos warrant further exploration.

## 6 RNA MODIFICATION IN MZT

*N*6‐methyladenosine (m^6^A) is the most abundant modification on RNA and is reversible in eukaryotic cells.^153^ Recently, it has been indicated that m^6^A is essential for maternal RNA degradation and early embryo development.^154^ In mice, Sui et al. found that the global RNA m^6^A level decreased from GV to 2‐cell stage but increased gradually after ZGA by immunofluorescence staining.^155^ Similarly, using ultralow‐input MeRIP‐seq technology, Wu et al. found that the global abundance of m^6^A decreased continuously during mouse MZT, but the number of m^6^A marked transcripts increased after fertilization (Figure 3).^156^ In GV and MII oocytes, 43% of maternal decay transcripts gained m^6^A, and m^6^A methylation was preferentially performed on Z‐decay than M‐decay transcripts. Moreover, m^6^A methylation likely promotes the transcription of Z‐decay transcripts. Indeed, m^6^A may also play an important role in ZGA. In late 1‐cell or late 2‐cell embryos, about 68% of ZGA transcripts are m^6^A modified, including transposons (like MERVL and MT‐int) and 2‐cell marker genes (like *Dux* and *Zscan4*).^156^ m^6^A methyltransferase METTL3 is important for both oocyte and embryo development. In mouse oocytes, knockout of *Mettl3* not only affects mRNAs degradation but also disrupts mRNAs translation efficiency, which lead to a low maturation rate.^155^ METTL3 is also essential for establishing m^6^A on ZGA transcripts and MERVL, and regulating the timely degradation of these transcripts.^155^, ^156^ As an m^6^A binding protein, YTHDF2 is abundant in oocytes and mediates RNA degradation process through recognizing and destabilizing m^6^A‐modified mRNAs.^157^ Although most *YTHDF2* deficiency oocytes can develop to MII stage, the derived embryos can develop beyond 2‐cell stage with the repressed maternal mRNAs decay.^157^ Indeed, according to single‐cell RNA‐seq data, YTHDF2‐deficiency mainly represses degradation of Z‐decay rather than M‐decay transcripts.^156^ Another m^6^A reader, IGF2BP2 is also required for early embryonic development.^158^ The deficiency of *IGFBP2* causes developmental arrest of majority of embryos at the 2‐cell stage and decreases both transcriptional and translational activity of transcriptions during ZGA.^158^ However, whether IGFBP2 regulates RNA degradation and ZGA though an m^6^A‐dependent manner remains to be explored.

## 7 NON‐HISTONE PROTEIN POST‐TRANSLATIONAL MODIFICATIONS IN MZT

Besides histone proteins, various studies have demonstrated that post‐translational modification of nonhistone proteins plays an important role in regulating the proper MZT.^3^, ^159^ For example, oocyte maturation requires correct phosphorylation and activation of MPF.^160^, ^161^ However, timely ubiquitination and degradation of MPF is responsible for the meiosis‐to‐mitosis transition upon fertilization. Here, we summarized recent discoveries regarding post‐translational modification of nonhistone proteins involved in MZT, including phosphorylation and ubiquitination.

### 7.1 Protein phosphorylation

Protein phosphorylation is an extensively studied post‐translational modification that involves in many biological processes in eukaryotes.^162^ Phosphorylation can alter the structure of substrate proteins by forming phosphoester bonds, thereby regulating the activation, localization, interaction, and stability of these proteins.^163^ In mammals, protein phosphorylation also participates in the processes of oocyte maturation and embryo development.^159^ It is reported that high level of activated MPF (cyclinB1/CDK1) is important for the initiation of oocyte meiotic resumption and the maintenance of MII arrest until fertilization.^161^ Moreover, the maintenance of oocyte MII arrest is essential for successful fertilization.^164^ Phosphorylated WEE1/MYT1 by PKA mediates that the phosphorylation of CDK1 at Thr14 and Tyr15 and is responsible for the maintenance of the inactive form of MPF. In humans, impaired phosphorylation of *Wee1b* (*Wee2*) caused by homozygous mutation of *Wee1b* is also found to be responsible for the occurrence of fertilization failure.^165^ Additionally, PKA phosphorylates CDC25 and causes its cytoplasmic localization that prevents the function of CDC25 to cleave the inhibitory phosphorylation and activate CDK1.^166^, ^167^ Contrary to PKA, SGK1 and AURKA have been demonstrated to be responsible for the activation of MPF by phosphorylating and activating CDC25.^168^, ^169^

Besides, ERK1/2‐mediated phosphorylation is also essential for oocytes oocyte meiotic maturation and MZT.^170^ ERK1/2 can promote the phosphorylation and activation of CPEB1, which triggers the polyadenylation and translation activation of the maternal mRNAs containing CPE in oocytes. Moreover, the translation products of ERK 1/2‐targeted mRNAs are required for the degradation of maternal mRNAs, such as BTG4 and CNOT7.^170^ Therefore, the dysregulation of some kinase or phosphatases might cause the abnormal development of early embryos by aberrant activation or repression of key genes during MZT.

### 7.2 Protein ubiquitination

Protein ubiquitination, an important post‐translational modification, is involved in ubiquitin‐proteasome pathway.^100^ The proteins destined for degradation are first tagged with ubiquitin protein through the sequential action of ubiquitin activating (E1), conjugating enzymes (E2), and ligases (E3) enzymes and finally degraded by the 26S proteasome.^171^ It has been reported that the ubiquitin‐proteasome pathway plays an important role in the degradation of maternal proteins after fertilization.^3^, ^172^ The result from MS analysis indicated that ubiquitination‐related components are highly upregulated in zygotes compared with oocytes.^7^ In mammals, matured oocytes are arrested at MII, and fertilization triggers the transition from meiosis to mitosis.^173^ E3 ligase APC/C and RFPL4 are reported to regulate MII exit by promoting the cyclin B degradation and resulting in MPF destabilization after fertilization.^160^ EMI2/XERP1 can inhibit the activity of APC/C, which plays an important role in the maintaining of oocyte MII arrest.^174^, ^175^ After fertilization, phosphorylated EMI2/XERP1 is recognized by SCF and promotes the ubiquitination and degradation of EMI2/XERP1. Activated APC/C working with its coactivator CDC20 mediates the degradation of cyclin B to facilitate the exit of MII.^176^, ^177^, ^178^ Furthermore, RFPl4 can also interact with cyclin B to promote its degradation.^160^ Recently, another E3 ligase RNF114 is also reported to be highly expressed in oocytes and early embryos.^179^ The knockout of *Rnf114* results in abnormal protein metabolism in oocytes, and most maternal KO embryos fail to develop beyond the 2‐cell stage with major ZGA defects.^179^, ^180^ In line with this, the knockdown of some RNF114 substrates, *Tab1* or *Cbx5*, can partially rescue the abnormal development of maternal *Rnf114* KO embryos.^179^, ^180^ The ubiquitination proteasome system is complicated and includes more than 600 E3 ligase and many deubiquitinases to maintain the protein abundance in a normal level to ensure the normal development of embryos and normal growth of cells. However, limited by the length of the paper, a plenty of E3 ligase or deubiquitinases that are responsible for the MZT or early development of embryos were not summarized and discussed which might warrant a through and detailed discussion in a separate review article.

## 8 CANDIDATE REGULATORS OF MZT

MZT involves maternal material decay and zygotic genomic activation, which is crucial for early embryo development.^2^ Some important factors of maternal mRNAs decay and the regulator pathways have been reported in recent years. However, the key regulators of ZGA initiation still remain elusive. Here, we reviewed the latest findings on the possible candidate regulators of maternal decay and ZGA initiation during MZT.

### 8.1 Regulators of maternal mRNAs degradation

**FIGURE 5:**
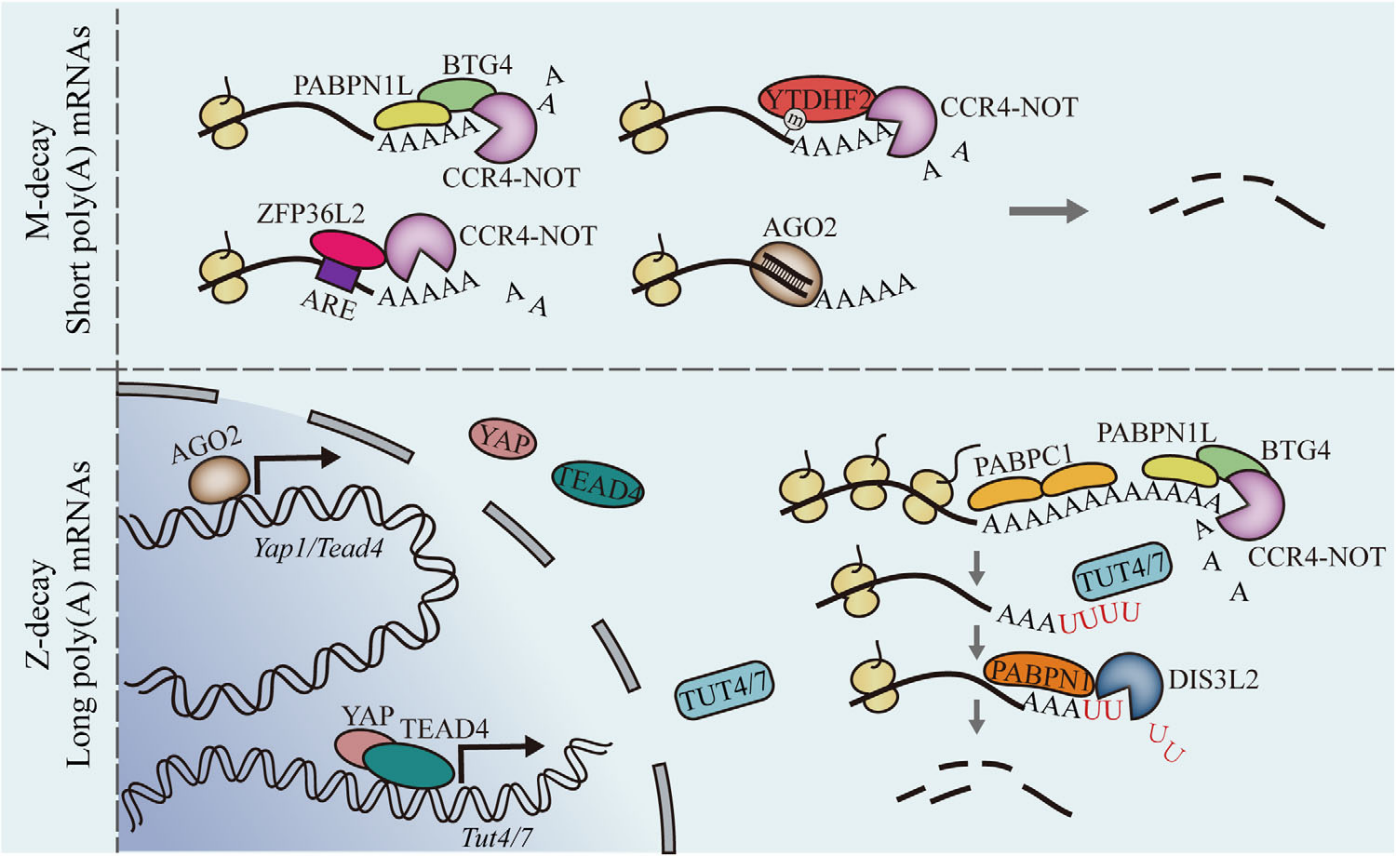
**An overview of regulators important for maternal transcriptome degradation during maternal‐to‐zygotic transition**. For M‐decay, maternal BTG4 can recruit CCR4‐NOT complex to mRNAs via interacting with the poly(A) binding proteins PABPN1L. ZFP36L2 can also recruit CCR4‐NOT complex to ARE‐containing mRNAs. m^6^A reader also can recruit CCR4‐NOT complex to mRNAs. AGO2 can bind to the mRNAs through endosiRNA. Maternal transcripts undergo Z‐decay tend to have longer 3′‐UTRs and higher translational activity than those undergo Z‐decay. BTG4‐CCR4‐NOT complex also mediated the deadenylation of Z‐decay transcripts and promote the dissociation of PABPC1 from mRNAs. TUT4/7 mediated the uridylation of mRNAs with short poly(A) tails (<25 nucleotides), and DIS3L2 which recruited by PABPN1 is important for the degradation of oligouridylated mRNAs.

In oocytes, numerous mRNAs are translationally repressed and stably accumulated in cytoplasm and will be recruited for translation during oocyte meiotic maturation and early embryonic development.^181^ In the meantime, timely degradation of translated maternal mRNAs is also important for the developmental control transferred from the maternal‐to‐zygotic genome.^4^ Here, we summarized the important regulators involved in the stabilization and degradation of maternal mRNAs during MZT (Figure 5).

#### 8.1.1 Stabilization of maternal mRNAs

Recently, Cheng et al. reported that the maternal mRNAs are stored in a mitochondria‐associated membranelles compartment (MARDO) in mammalian full‐grown oocytes. RNA‐binding proteins like ZAR1, MSY2, DDX6, LSM14B, and 4E‐T are localized in MARDO. Moreover, ZAR1 is essential for the assembly and coalescence of the MARDO around mitochondria. *ZAR1* deficiency causes delayed GVBD and PB1 emission in oocytes, with the disruption of MARDO and the premature loss of mRNAs that localized in MARDO.^182^ Ultimately, embryos derived from *ZAR1* null oocytes are blocked at 1‐cell stage embryos, resulting in the infertility of *ZAR1* maternal KO female mice.^183^ *Zar2* is the homolog of *Zar1*, which is dispensable for the development of oocyte and female fertility. However, the double deletion of both *ZAR1* and *ZAR2* leads to a severer phenotype than *ZAR1* deletion, which indicating that ZAR2 seems to enhance the function of ZAR1. *ZAR1/2* knockout oocytes are primarily arrested at MI stage with impaired mitotic resumption and emission of PB1 and spindle assembly defects, implicating maternal mRNA instability and impaired translation of important oocyte proteins. Moreover, *ZAR1/2* maternal deficiency embryos also fail to develop beyond the 2‐cell stage.^183^ Besides maintaining global mRNA stability in GV oocytes, the physical binding of ZAR1/2 to mRNAs also facilitates the translation of important oocyte proteins after meiotic resumption, such as BTG4, WEE2, and TPX2.^183^

#### 8.1.2 Degradation of maternal mRNAs controlled by the maternal transcriptome

It is reported that maternal‐factor BTG4 is essential for M‐decay in mouse oocytes and zygotes. Although the knockout of *Btg*4 does not affect the maturation of oocytes, embryos derived from *Btg*4^−/−^ females are arrested at the 1‐ or 2‐cell stage with M‐decay defect.^184^, ^185^ BTG4 regulates mRNAs decay by recruiting the CCR4‐NOT complex (binds CNOT7/8 subunit) to target M‐decay transcripts and facilitate the deadenylation of these transcripts.^184^, ^185^ Moreover, PABPN1L binding to the poly(A) tail of mRNAs is required for the recruitment of BTG4.^186^ The maternal depletion of *Pabpn1l* also causes the embryo arrest at the 1‐ or 2‐cell stage.^186^ Importantly, BTG4 is also required for the normal development of human embryos. The homozygous mutation in *Btg4* causes zygotic cleavage failure and impairs the degradation of maternal transcripts in human embryos.^187^ Moreover, the ERK1/2 signal pathway plays a pivotal role in the translation of BTG4 and CNOT7 by activating CPEB1 in mouse oocytes.^170^, ^188^ Biallelic mutations of *Mos*, which encodes the upstream kinase of ERK1/2, also disrupts maternal mRNA clearance in human oocytes, and causes female infertility, characterized by human early embryonic arrest.^188^

Another RNA binding protein ZFP36L2 can bind ARE‐harboring maternal mRNAs (A/U‐rich element) and recruit the CCR4‐NOT complex (binds CNOT6L subunit) to promote the degradation of these mRNAs during meiotic maturation in mouse oocytes. The degradation mediated by ZFP36L2‐CCR4‐NOT^CNOT6L^ occurs earlier and in a wider range than that triggered by BTG4‐CCR4‐NOT^CNOT7/8^.^189^, ^190^ Over‐translation of these insufficiently degraded mRNAs leads to the activation of spindle assembly checkpoint that causes the MI arrest of *CNOT6L* ^−/−^ oocytes. Consequently, majority of zygotes derived from these oocytes failed to develop beyond the 4‐cell stage.^189^, ^190^ Moreover, ZFP36L2‐CCR4‐NOT^CNOT6L^ guided maternal mRNA decay has been demonstrated to be necessary for accurate MZT in human. The biallelic mutation in *Zfp36l2* perturbs the removal of massive ARE‐containing transcripts in human zygotes and is potentially responsible for the occurrence recurrent preimplantation embryo developmental arrest.^191^ Altogether, the stage‐specific maternal mRNAs decay guided by these different modes of CCR4‐NOT recruitment is conserved in mammals. Notably, the RNA m^6^A modification is important for maternal mRNAs degradation during MZT.^192^ The RNA m^6^A reader YTHDF2 mediates the mRNAs degradation by recruiting CCR4–NOT complex (binds CNOT1 subunit) in mammalian cells.^193^

#### 8.1.3 Degradation of maternal mRNAs controlled by the zygotic transcriptome

Z‐decay transcripts tend to have longer 3′‐UTRs and higher translational activity than M‐decay transcripts in matured oocytes.^6^ BTG4–CCR4–NOT complex is also involved in Z‐decay by mediating the deadenylation of Z‐decay transcripts and providing the ideal targets for TUT4/7.^6^, ^194^, ^195^, ^196^ TUT4/7 can catalyze the 3′ terminal uridylation of mRNAs with short poly(A) tails (<25 nucleotides) that lost the stabilizing PABPC1 binding, which is required for oocyte growth.^197^ Moreover, it is found that the loss of BTG4, CNOT7, or TUT4/7 in zygote impaired several Z‐decay mRNAs degradation.^6^ Maternal YAP1 and zygotic TEAD4 can activate the zygotic expression of *Tut4/7*.^6^, ^194^ Upon 3′‐uridylation catalyzed by TUT4/7, PABPN1 can dock on 3′‐uridylated Z‐decay transcripts and recruit 3′–5′ exoribonuclease DIS3L2 to facilitate the progression of Z‐decay transcripts degradation.^196^ Besides, *Ago2* is reported to be highly expressed in mouse oocytes and early embryos. The deletion of *Ago2* in mouse zygotes impairs the development of early embryos and the degradation of over half of the maternal mRNAs.^198^ AGO2 can target the maternal mRNAs through endogenous small interfering RNAs (endosiRNA) and cooperate with P‐bodies to facilitate the maternal mRNAs decay. Moreover, the digestion of dsRNAs generated by the bind of maternal RNAs with their complementary long noncoding RNAs (CMR‐lncRNAs) is important for AGO2‐dependent maternal mRNAs degradation. AGO2 is also involved in the Z‐decay through acting with small acting RNAs to active the zygotic expression of YAP1 and TEAD4.^198^ In human, compared to M‐decay transcripts, Z‐decay transcripts also have longer 3′‐UTRs.^8^ Similar with that in mouse embryos, the inhibition of YAP‐TEAD4 leads to the reduction of TUT4/7 and accumulation of Z‐decay transcripts in human PN3 embryos.^8^ These results indicated that the mechanism of Z‐decay may be conserved between mouse and human embryos.

### 8.2 Regulators of zygotic genome activation initiation

To date, the key factors required for ZGA initiation in mammal embryos are largely unknown. Pioneer factors are a class of transcript factors (TFs), which can bind near chromatin directly and subsequently initiate gene expression.^199^ The pioneer factor Zelda is widely known to be essential for the zygotic gene activation in Drosophila.^200^ Whether pioneer factors awake the mammalian embryonic genome remains largely unknown. One study in mice found that NFYA as a pioneer factor is required for a subset of ZGA gene activation in 2‐cell embryos.^201^ The maternal deficiency of NFYA leads to a considerable decrease of DNase I‐hypersensitive site (DHSs) in promoters at the 2‐cell stage and causes the developmental arrest of majority of embryos at the morula stage. Moreover, a significant reduction of DHSs signals preferentially occurs in genes containing NFYA binding motifs rather than others.^201^ Moreover, NFYA deficiency impairs the major ZGA with a largely decreased signal of DHSs in promoters of downregulated ZGA genes. However, the fact that only 15% of major ZGA genes are downregulated in NFYA‐deficiency 2‐cell embryos suggests the existence of other TFs responsible for ZGA.^201^

Recently, maternally inherited orphan nuclear receptor NR5A2 is reported to be an important pioneer TF for genome‐wide gene activation during ZGA in mouse embryos.^202^ Chemical inhibition of NR5A2 impairs embryo developmental potential and leads to the downregulation of 72% of ZGA genes as well as significant decrease of chromatin accessibility at where it binds.^202^ NR5A2 mainly targets the SINE B1/Alu retrotransposons that are much close to the TSS of ZGA genes rather than non‐ZGA genes. When NR5A2 binds to these elements that close to the TSS of the ZGA genes, it can promote the transcriptional activation of these genes during ZGA.^202^ Besides the retrotransposons, NR5A2 can also bind to the enhancer like sequences (high H3K27ac and ATAC‐seq and low H3K4me3 signal). Moreover the ability of NR5A2 binding to nucleosomal DNA in vitro indicated that NR5A2 can function as a pioneer factor. Another orphan nuclear receptor ESRRB also was involved in ZGA as a pioneer TF at the 2‐cell stage.^202^ Whether NR5A2 directly controls most ZGA genes activation or indirectly functions with other pioneer TFs like ESRRB remains unknown.

**FIGURE 6:**
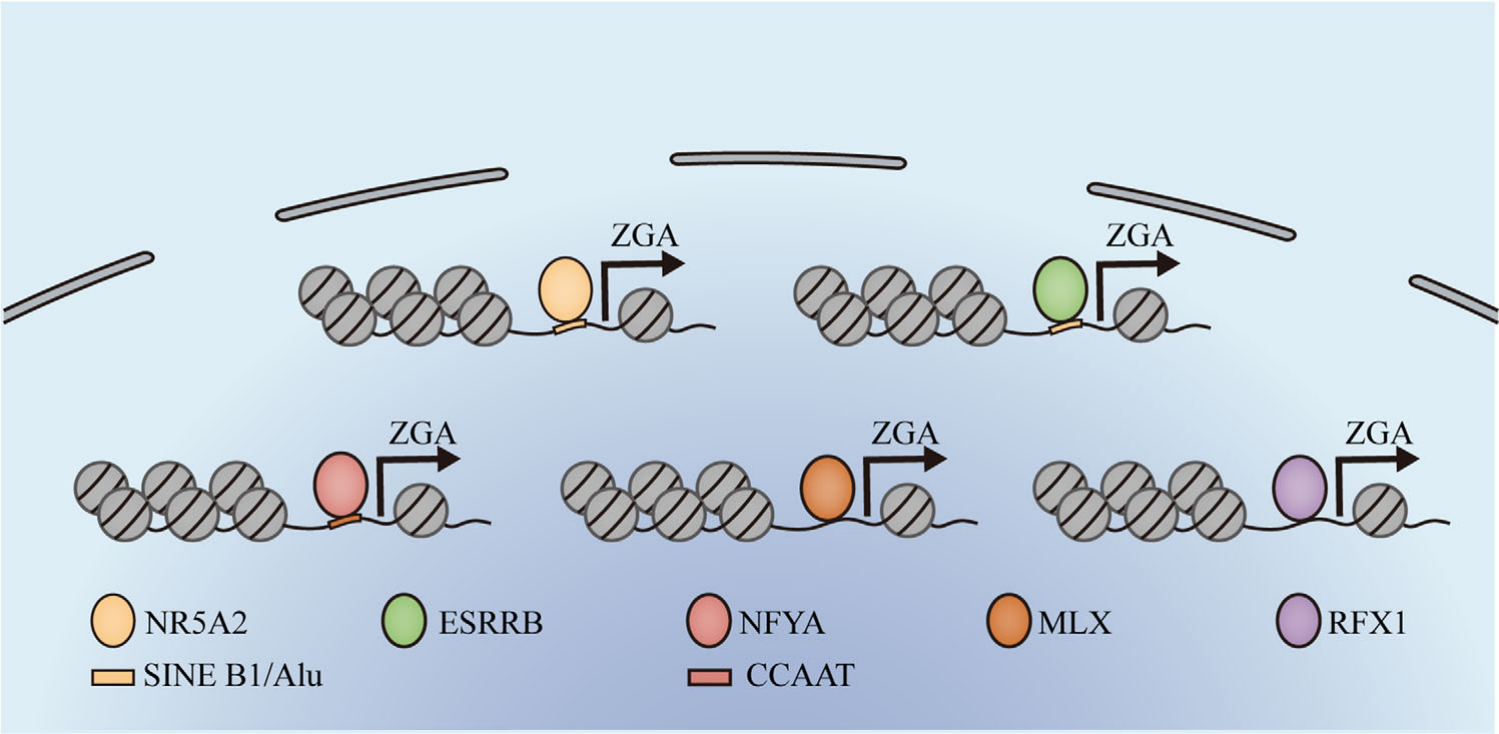
**An overview of pioneer transcript factors important for zygotic genome activation (ZGA) initiation**. NR5A2 and ESRRB can bind to the SINE B1/Alu retrotransposons to increase chromatin accessibility and regulate ZGA genes expression. NFYA can bind to CCAAT motif to increase chromatin accessibility and regulate ZGA gene expression. MLX and RFX1 play important roles in nucleosome‐depleted region (NDR) formation to regulate ZGA gene expression.

Another report indicated that MLX and RFX1 were important for the nucleosome‐depleted regions formation in promoters of minor or major ZGA genes in the paternal pronuclei of the mouse ICSI embryos.^203^ MLX binding motifs are enriched in promoters of minor ZGA genes, and RFX1 binding motifs are enriched in promoters of major ZGA genes. The knockdown of *Mlx* or *Rfx1* leads to significant downregulation of minor or major ZGA genes, respectively.^203^ Furthermore, the homozygous knockout of *Rfx1* causes embryo lethality before the morula stage.^204^ Moreover, the major ZGA genes regulated by NFYA have few overlaps with those regulated by RFX1, which suggested that the regulation of ZGA may require the cooperation of multiple TFs (Figure 6).^203^

Besides, DUX and its human ortholog DUX4 are found to be expressed before major ZGA in mouse or human embryos, respectively.^205^ Overexpression of *Dux4* in human induced pluripotent stem cells or *Dux* in mESCs can activate the transcription of cleavage‐specific genes and ERVL‐family retrotransposons.^206^ Additionally, Dux expression is both necessary and sufficient for the transition from mESCs to two‐cell embryo‐like (2C‐like) cells.^206^, ^207^ However, contrary to what was observed in cells, loss of DUX in mouse embryos only decreases the expression of a small subset of ZGA genes.^208^, ^209^ More importantly, *Dux* homozygous knockout mice can survival to adulthood, which further indicates that DUX does not play a major role in ZGA initiation in mouse embryos.^208^, ^209^ But the timely *Dux* silencing at DNA level as well as the degradation at RNA and protein levels are necessary for the exit of 2‐cell state and embryo development.^209^, ^210^, ^211^

## 9 PERSPECTIVES

In this review, we comprehensively summarized the current knowledge of epigenetic reprogramming during MZT. During MZT, embryos undergo temporally controlled maternal decay and zygote genome activation, with dramatic global epigenetic reprogramming.^212^, ^213^ Although there are many studies about epigenetic remodeling during this process, most of them are descriptive and the precise mechanisms are still largely unknown. The key factors and molecular mechanisms of the reprogramming of epigenome at different genome loci were not well discussed. For example, it is largely unknown how ncH3K27ac is established. In mouse and human embryos, both H3K4me3 and H3K27ac domains exist as a noncanonical pattern and highly overlapped before ZGA. Thus, it is possible that the establishment of these two modifications is mediated by an epigenetic complex.^130^

Accumulative evidences indicated that different types of epigenetic modifications could work with each other. It has been found that H3K4me3 is positively correlated with DNA methylation by repelling DNA methyltransferases, whereas H3K9 methylation in the maternal pronuclei protects maternal genome from TET3‐mediated DNA demethylation.^75^, ^102^, ^125^ In oocytes, DNA methylation on gene bodies of transcribed genes can protect these regions from the binding of PRC1/2, preventing the deposition of H3K27me3/H2AK119ub, thus ensuring the normal transcription of certain genes.^107^, ^214^ In *CXXC1*‐depleted mouse oocytes, H3K27me3/H2AK119ub1 is increased mainly in gene body regions where DNA methylation was significantly decreased.^96^ Whether H3K27me3/H2AK119ub1 can in turn inhibit DNA methylation is still unknown and needs to be further explored. A recent study also found that H3K9me3 prevented the loss of TADs at the 2‐cell stage SCNT embryos.^123^ The interplay among different epigenetic modifications may be essential for the precise regulation of MZT, and the detailed mechanisms remain largely unexplored.

Furthermore, the relationship between epigenome reprogramming and ZGA remains unclear. Several reports proposed that the clearance of H3K27me3 and the establishment of higher order chromatin conformation can occur without transcriptional activation of the zygotic genome.^107^, ^134^, ^137^ However, the transition from ncH3K4me3 to canonical H3K4me3 depends on ZGA.^73^ Further multi‐omics studies are required to disentangle the precise crosstalk between epigenome and transcriptome in early embryos. The advanced low‐input epigenomic profiling technologies can be helpful to probe epigenetic reprogramming landscape during early embryo development. Further studies are required to clarify the detailed epigenetic regulatory network during MZT, and the results of which will help us to discover novel strategies to treat infertility and other diseases.

## AUTHOR CONTRIBUTIONS

Xiangpeng Dai and Yurong Chen conceived the topic and Yurong Chen wrote the manuscript with part help from Luyao Wang and Fucheng Guo. Xiaoling Zhang and Xiangpeng Dai edited the manuscript. All authors read and approved the final manuscript.

## CONFLICT OF INTEREST STATEMENT

The authors declare that they have no conflicts of interest.

## ETHICS STATEMENT

Not applicable.

## Data Availability

Not applicable.
